# A Conversation with Leo Gross

**DOI:** 10.1021/acscentsci.3c00636

**Published:** 2023-06-02

**Authors:** Payal Dhar

Leo Gross is a physicist who has devoted his career to studying the fundamental secrets of chemistry, that is, how atoms and molecules behave and interact with one another. As leader of IBM’s atom and molecule manipulation group in Zurich, Gross says his tools of choice are the scanning tunneling microscope (STM) and atomic force microscope (AFM).

**Figure:**
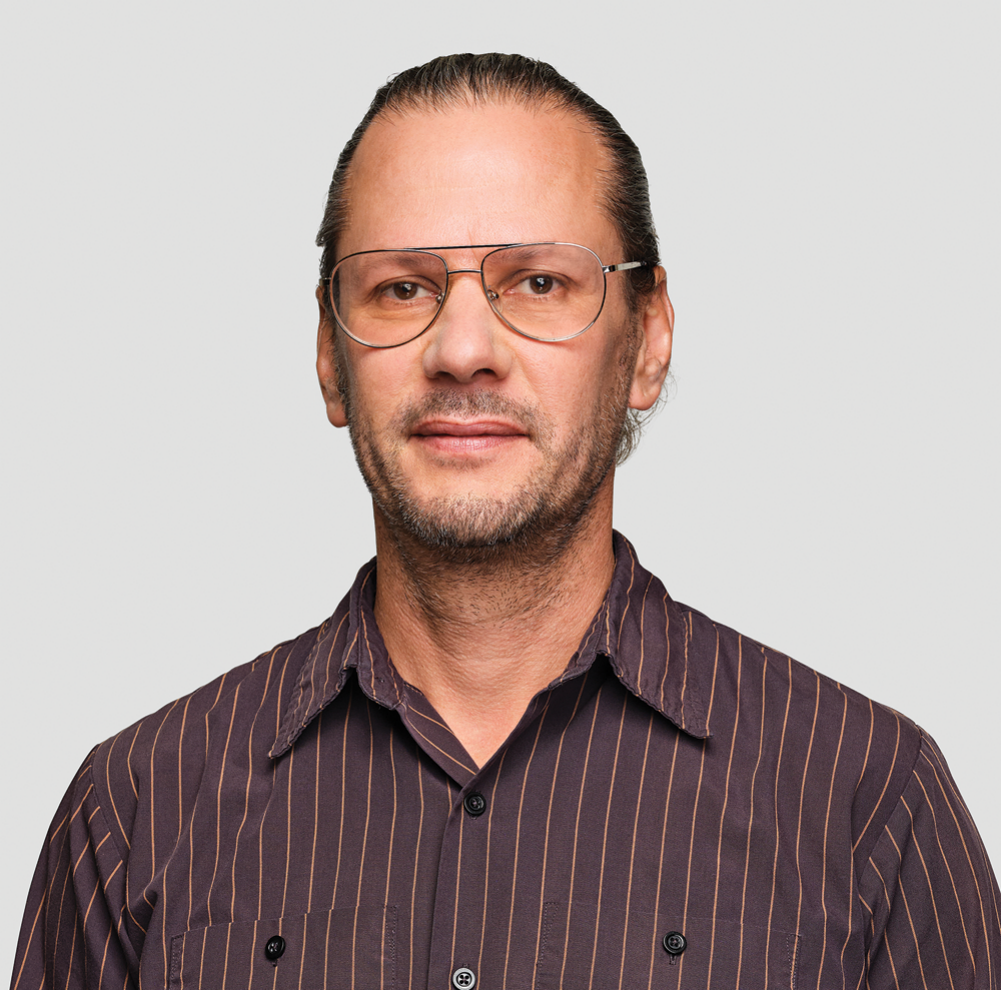
Credit: IBM Research

Both STMs and AFMs have ultrasharp tips that map atomic-scale surfaces, the former by emitting voltage pulses and the latter by sensing the electronic forces associated with individual atoms as the tip hangs just nanometers above a sample. But Gross’s team goes a step further, using STMs and AFMs to not just observe atoms but trigger and then monitor highly controlled single-molecule reactions. Last year, the group published a study showing that it was possible to select which reactions would take place in a single molecule using an STM tip. The discovery was featured on the cover of *Science*.

Payal Dhar spoke to Gross about what information chemists can glean from looking at individual molecules and the potential applications of his work at IBM.

## How did a physicist like you end up working on single-molecule reactions and molecular manipulation?

I wouldn’t make a clear distinction between chemistry and physics. These things are overlapping. I still do both.

I went for an exchange year to the U.S. during my undergraduate studies, where I worked in a group led by Ulrike Diebold at Tulane University. We used scanning tunneling microscopy to look at titanium dioxide. I liked that—it was so interactive. You got a real image immediately; you could change the parameters, do something with the tip, and directly change things on the surface.

You can say it is a little bit like playing a computer game, but it’s happening for real, just on the atomic scale. I was hooked; and later, at the universities where I studied and the positions I got, I sought out groups using these techniques.

## What are single-molecule reactions, and why are they important?

A single-molecule reaction is when we follow the reactions of a particular molecule with atomic-scale resolution. We do these reactions with the tip of the microscope, applying voltage pulses, with which we can break bonds or sometimes make bonds.

What we are interested in is really fundamental questions about what’s going on at the atomic scale when a reaction takes place. In single-molecule reactions, we can see the structure of the molecule using an AFM. We can actually see where the bonds are, get some information about bond order, and see how strong a bond is. With an STM, we see the orbital densities. You might remember them being shown as lobes in your chemistry textbook.

And from this, we can get a very detailed picture of the molecule, the reaction products, and the reaction itself. This gives us unprecedented insight about how these reactions are triggered, how they’re happening, why we get this selectivity, and what parameters play a role—insights that we cannot obtain in conventional synthesis.

**Figure:**
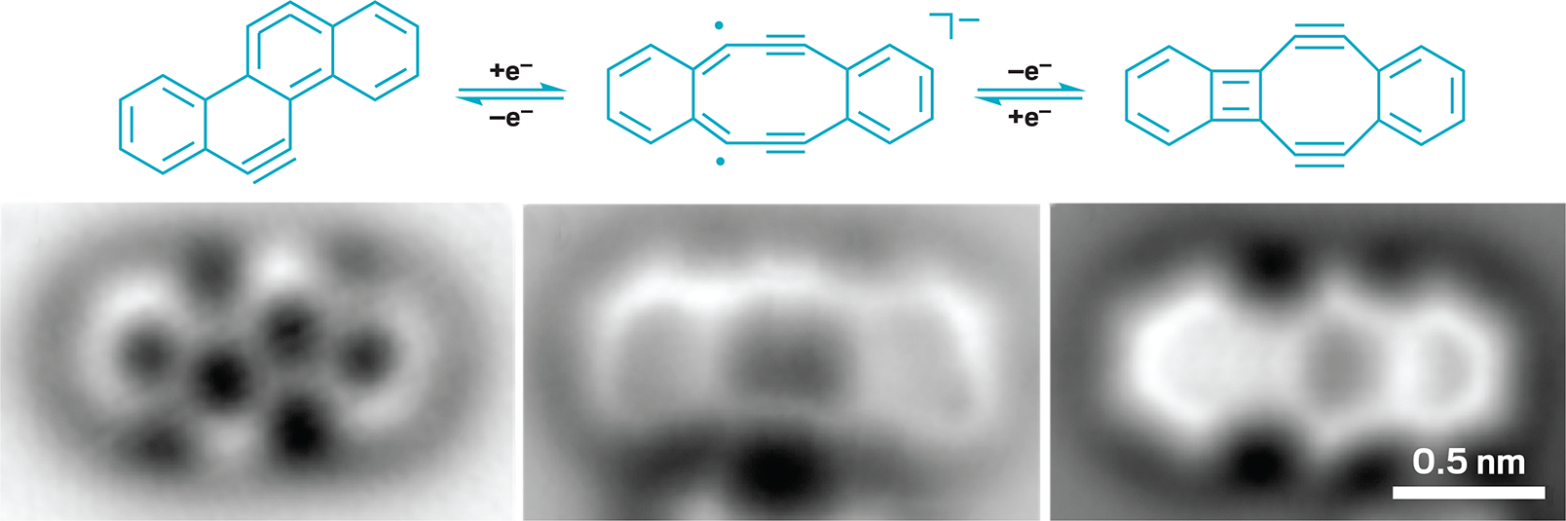
By changing the voltage at the tip of their scanning tunneling microscope, Gross and team could pluck off or add electrons to a molecule. Then, using an atomic force microscope, they observed which molecule they had produced (bottom row). Credit: *Science* (micrographs).

## What led you to investigate selectivity in tip-induced single-molecule reactions? What did you find?

We intended to make a molecule that had a big open ring in the center. Instead, we ended up with one of three molecules. Depending on the value of the voltage we applied at the tip, we can choose which one to make. This was very unexpected because with tip-induced reactions we usually cleave the bond that is the weakest and make one certain bond. The other thing is that because it is so controllable, we can learn a lot about the reaction because we can repeat it hundreds of times.

By the way, it was a collaboration; we worked with Diego Peña Gil at the University of Santiago de Compostela.

## What kind of applications would there be for this kind of molecular manipulation and reaction monitoring? Can they be useful on a large scale?

Controlling selectivity in a reaction could be interesting in molecular machines, let’s say, but it’s very fundamental.

Then, we also apply AFM and STM techniques as analytical tools, like for analysis of chemicals. With conventional methods, you need a certain amount of material to analyze, but with our technique, you can look at individual molecules in complex mixtures. At the moment, we are studying molecules formed in combustion engines—how soot particles form in a flame using single-molecule methods. We won’t clean up combustion with our microscopes, but we bring in some understanding on the atomic scale, and often this has implications on very big things, like health or climate.

We are not after a certain application. We will not use single-molecule reactions with an STM and AFM to produce molecules on an industrial scale. We are studying this model system to learn something, but this will not be used for, like—I don’t know—taking 1000 microscopes and employing 1000 Ph.D. students to each make one molecule a day.

## If there’s no clear application, why has IBM—a technology company—been so interested in atomic and molecular manipulation research for such a long time?

I mean, we do make molecules with it, and they have been applied in research and development for IBM products. But IBM invented these microscopes for fundamental research [in the 1980s], and it still pursues this kind of fundamental research. It could be interesting in the far future when we think about using single molecules as elements in, for example, logic devices, but that’s very far out.

## What are the biggest challenges at the moment?

It would be supercool to add time resolution to single-molecule reactions—to follow reactions in time and take movies in a stroboscopic fashion. We are working with Jascha Repp’s group in Regensburg on this. He’s very good at doing that, combining ultrafast laser pulses with STM.

We also want to increase the versatility of the reactions to more complex molecules. We want even better control so that we can decide what molecules to make, how to make them with a high yield, and then make larger structures. There are interesting molecules that we are still trying to make, molecules that are big challenges for chemists, molecules they have tried to synthesize for decades. Now, using this tip to make bonds, we can synthesize several of these.

## Payal Dhar is a freelance contributor to

Chemical & Engineering News, *an independent news outlet of the American Chemical Society. This interview has been edited for length and clarity.*

